# A supercritical fluid workflow for the quality assessment of herbal drugs and commercial preparations from *Rhodiola rosea*

**DOI:** 10.1002/pca.3040

**Published:** 2021-02-26

**Authors:** Julia Langeder, Ulrike Grienke

**Affiliations:** ^1^ Department of Pharmacognosy University of Vienna Vienna Austria

**Keywords:** commercial product, herbal drug, quality control, *Rhodiola rosea*, SFC, SFE

## Abstract

**Introduction:**

Preparations from the *Rhodiola rosea* are experiencing an increase in popularity: extracts of dried roots and rhizomes are used as adaptogen to treat stress, fatigue, and weakness. To meet high pharmaceutical standards, fast and reliable methods to assess phytochemical variations in respect of quality control are needed.

**Objective:**

The aim of this study was to extract and quantify seven characteristic secondary metabolites of *R. rosea* , namely *p*‐tyrosol (**1**), rosin (**2**), rosiridin (**3**), salidroside (**4**), rosarin (**5**), rosavin (**6**), and tricin‐5‐*O*‐β‐d‐glucopyranoside (**7**) in 24 herbal drugs and seven commercial preparations using a newly established supercritical fluid workflow.

**Methods:**

The developed protocol allowed for an exhaustive extraction of compounds **1**–**7** using 60% carbon dioxide (CO_2_) and 40% methanol. The constituents were analysed on an ultra‐high‐performance supercritical fluid chromatography (UHPSFC) instrument using a charged surface hybrid fluoro‐phenyl (CSH FP) column (3.0 mm × 100 mm, 1.7 μm; mobile phase: CO_2_ and methanol).

**Results:**

The seven compounds were separated in a remarkably short time (< 3.5 minutes). For their quantitation, good results in terms of selectivity, linearity (*R* ^2^ ≥ 0.99), precision (intraday ≤ 3.03%, interday ≤ 5.17%) and accuracy (recovery rates 96.6–102.4%) were achieved using selected ion recording on a Quadrupole Dalton (QDa) mass detector.

**Conclusion:**

The quantitative analysis of the investigated herbal drugs showed a highly differing metabolite pattern which was also observed in the investigated commercial products. None of the commercial dietary products met the declared content of rosavins and salidroside. The developed and validated protocol offers a novel and reliable method to assess the quantitative composition of *Rhodiola* herbal drugs and preparations.

## Abstract 2

Hydroethanolic extracts of the dried roots and rhizomes of *Rhodiola rosea* are experiencing an increase in popularity, therefore methods for quantitative analyses are urgently needed regarding quality control. For the first time, a supercritical fluid workflow was applied to the polar secondary metabolites of rose root. Supercritical fluid extraction, followed by ultra‐high‐performance supercritical fluid chromatography allowed for the quantitation of seven compounds in 31 samples, not only commercial products but also herbal drugs.

## 1 INTRODUCTION

*Rhodiola rosea* L. (rose root, Arctic root or golden root), a species mainly growing in Arctic regions of Europe and Asia, has been a valuable medicinal plant used as adaptogen for centuries.^1^ The scientific evidence for the health beneficial properties such as anti‐depressive, anti‐fatigue, anxiolytic, cardioprotective, central nervous system (CNS) stimulating, neuroprotective, and nootropic effects is increasing continuously.^1^, ^2^ For commercial products including herbal medicinal products and dietary supplements in Europe, Asia, and the United States,^3^ mainly hydroethanolic extracts of rose root are used. The ever‐growing demand for raw plant material is provoking cases of adulteration with other plant species, low quality of the herbal material, as well as scarcity and endangerment of *Rhodiola* species.^4^, ^5^ To cover the subject of adulterations, Booker *et al*. established a combined platform based on spectroscopic [proton nuclear magnetic resonance (^1^H NMR) metabolomics] and chromatographic [high‐performance thin‐layer chromatography (HPTLC)] methods to distinguish five different species of *Rhodiola*.^5^ This platform was also applied to unregistered dietary supplements with alarming results: About 25% of these products were either adulterated or did not conform to their label specification regarding rosavin levels.^6^

The United States Pharmacopoeia (USP) contains a monograph for *R. rosea* as well as for the.

Asian species *R. crenulata* (Hook.f. & Thomson) H.Ohba,^7^, ^8^, ^9^ whereas the Chinese Pharmacopoeia only records *R. crenulata* ^10^ and the Russian Pharmacopoeia only *R. rosea*.^11^, ^12^ The elaboration of a monograph for the European Pharmacopoeia is still pending.^13^

Roots and rhizomes of rose root mainly contain phenylethanoids, phenylpropanoids, monoterpene alcohols, flavonoids, and their respective glycosides as well as proanthocyanidins and gallic acid derivatives.^1^ In dried roots and rhizomes, the USP monograph requires not less than 0.08% salidroside and not less than 0.3% phenylpropanoids (comprising rosavin, rosarin, and rosin),^7^ whereas the Russian Pharmacopoeia demands a more than three‐times higher content of phenylpropanoids (> 1.0%) and a 10‐times higher content of phenylethanoids calculated as salidroside (> 0.8%) (Table 1).^12^

**TABLE 1:** Requirements of different Pharmacopoeias for monographed *Rhodiola rosea* and *R. crenulata* roots and rhizomes

Required content of rosavins^a^	Required content of saildroside	Monographed *s*pecies	Pharmacopoeia/source
>1.0%	>0.8%	*R. rosea*	State Pharmacopoeia of the Russian Federation^12^
>0.3%	>0.08%	*R. rosea*	United States Pharmacopoeia^9^
>1.0%	>0.6%	*R. crenulata*	
n.g.^b^	>0.5%	*R. crenulata*	Pharmacopoeia of the People's Republic of China^10^

^a^ Calculated sum of cinnamyl alcohol derivatives rosavin, rosarin and rosin.

^b^ Not given.

Regarding quality and sustainability, Peschel *et al*. recently analysed the influence of geographic provenance, harvest season, plant sex, plant part (root or rhizome), and processing on marker compounds of *Rhodiola* species.^14^ Especially, marker ratios, e.g. salidroside vs. total rosavin content or rosarin vs. rosavin vs. rosin, turned out to be useful for quality control. Whether the plant is male or female showed no influence on the phenylpropanoid content^15^ and also drying temperature and cutting conditions are less important.^14^ However, origin, harvest season, plant part (rhizomes contain more rosavins than roots), and processing have a major influence on the quantity of *Rhodiola* constituents.^15^

Commonly applied extraction procedures for rose root samples comprise sonication and maceration using solvents such as methanol or ethanol or hydroethanolic/methanolic mixtures ranging from 38% to 75% alcohol.^16^, ^17^, ^18^, ^19^, ^20^, ^21^, ^22^ Moreover, accelerated solvent extraction with 85% methanol and 15% water was reported.^19^

Classic chromatographic approaches to study phytochemical variations in different *Rhodiola* samples most commonly involve a high‐performance liquid chromatography (HPLC) instrument hyphenated to an ultraviolet‐visible (UV‐vis) detector.^16^, ^17^, ^18^, ^20^, ^23^ However, up until now, published protocols are suffering from drawbacks, e.g. long analysis times of more than 30 minutes^16^, ^19^, ^21^, ^22^, ^23^ or they are limited to the analysis of only one or few compound classes.^18^, ^20^, ^24^

Supercritical fluid‐based (SFx) technologies have many advantages for the extraction and separation of plant constituents, e.g. little solvent consumption and remarkable short analysis times for high efficiency separations due to the high diffusivity and low viscosity of the mobile phase. Due to the non‐polar character of supercritical carbon dioxide (CO_2_), the primary focus of separation lies on non‐polar analytes like carotenoids, fatty acids or terpenes.^25^ However, the adjustment of the mobile phase polarity with organic modifiers and the availability of new stationary phase materials with sub‐2 μm particles extend the spectrum of this technology. Thus, also polar compounds like glycosides can be extracted and separated successfully.^26^ For instance, Gibitz‐Eisath *et al*. recently achieved the separation of seven glycosides to establish a quantitation method for common vervain.^27^

**FIGURE 1:**
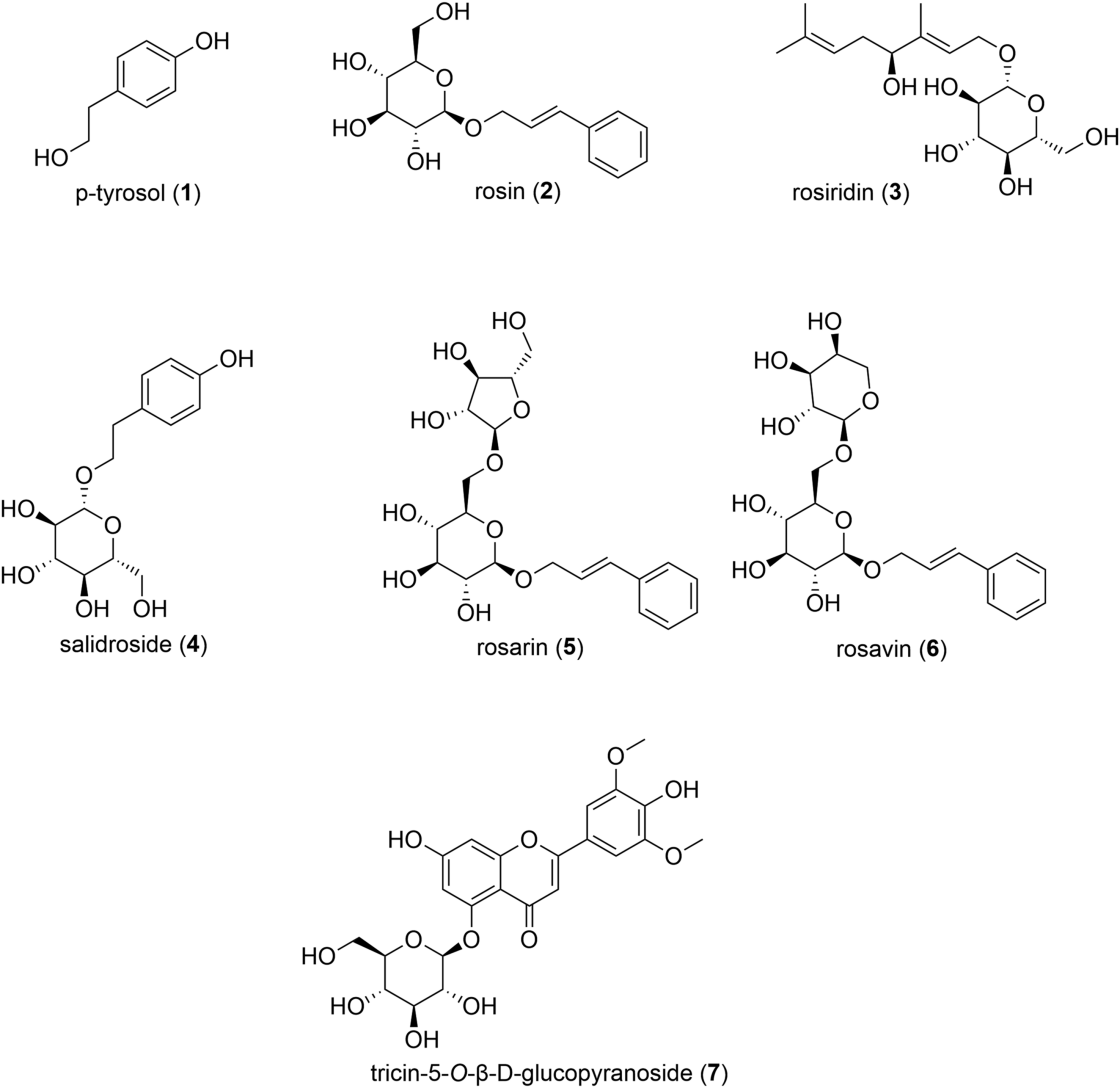
Chemical structures of compounds **1**–**7**

The aim of this study was to overcome disadvantages of current standard protocols for the extraction and quantitation of characteristic *R. rosea* constituents. This goal was implemented with a fast, ecofriendly and efficient workflow taking advantage of CO_2_ based technologies. Whilst therapeutic effects of *Rhodiola* constituents are still the subject of intense research, phenylpropenoid glycosides, i.e. rosin (**2**), rosarin (**5**), and rosavin (**6**), the monoterpene glucoside rosiridin (**3**) as well as the phenylethanoid *p*‐tyrosol (**1**) and its glucoside salidroside (**4**) are well established marker compounds (Figure 1). Although tricin‐5‐*O*‐β‐d‐glucopyranoside (**7**) has not been considered in previous standardisation studies of *Rhodiola*, it was included in the present study for two reasons: (i) for its importance as anti‐influenza A virus active,^28^ α‐amylase^29^ and NO production inhibiting^30^ compound and (ii) due to its polarity which increased the challenge for the development of SFx based protocols for extraction and separation. The established methods were validated and applied for the quantitation of **1** to **7** in 31 *R. rosea* samples (commercial products as well as herbal drugs).

## 2 EXPERIMENTAL

### 2.1 Samples and reagents

Previously, seven constituents from *R. rosea* roots and rhizomes were isolated from a standardised 70% ethanolic extract (SHR‐5): *p*‐tyrosol (**1**), rosin (**2**), rosiridin (**3**), salidroside (**4**), rosarin (**5**), rosavin (**6**), and tricin‐5‐*O*‐β‐d‐glucopyranoside (**7**).^28^ For isolation procedures, the dry powder of the SHR‐5 extract (batch no. 1521229, Voucher specimen JR‐20180904‐A1 deposited at the Department of Pharmacognosy, University of Vienna, Austria) was applied to high‐performance counter current chromatography gradient elution.^28^ Isolated compounds were identified by the interpretation of one‐dimensional (1D) and two‐dimensional (2D) NMR and electrospray ionisation‐mass spectrometry (ESI‐MS) experiments. Purities were determined using an ultra‐performance liquid chromatography‐evaporative light scattering detector (UPLC‐ELSD):^28^ 98% (**1**), 99% (**2**), 98% (**3**), 95% (**4**), 97% (**5**), 96% (**6**) and 94% (**7**), respectively.

Between February and July 2020, two approved herbal medicinal products (RR01 and RR02, coated tablets), five dietary supplements (RR03–RR07, capsules) containing rose root extracts were purchased from online pharmacies in Austria and Germany. Additionally, 24 herbal drugs (raw material samples; not authenticated) sold as *R. rosea* root and/or rhizome (RR08–RR31) were obtained from different plant cultivation companies in Europe, Asia, and North America. Voucher specimens of all samples are deposited at the Department of Pharmacognosy, University of Vienna, Austria (Supporting Information Table S2).

Before extraction, crude root samples were ground to powder using a household grinder. All powder samples were kept in paper bags at room temperature until use. All HPLC grade solvents were purchased from VWR Chemicals. Compressed 4.5 grade CO_2_ (purity ≥ 99.995%) was purchased from Messer.

### 2.2 Supercritical fluid extraction

Extractions were performed using a Waters MV‐10 supercritical fluid extraction (SFE) instrumentation consisting of a fluid delivery module (cooled down by a Thermo Scientific Accel 500 LC chiller), a 10 vessel column oven, an automated backpressure regulator, a heat exchanger and an extraction collector connected to a make‐up pump. The instrument is controlled via ChromScope 1.6 software. The extraction vessels hold a volume of 5 mL each. Regarding the commercial rose root products, the content of one capsule or one mortared tablet (except for RR06: two capsules) was placed into the extraction vessel and filled up with glass beads. Concerning the herbal drugs, 1.00 g of ground and dried sample was placed in the extraction vessel and filled up with glass beads. The optimised extraction method is presented in Table 2. The obtained extracts were transferred into a volumetric flask and filled up with methanol to 250.0 mL. The established protocol provides an extraction efficiency of more than 96.0% for all reference compounds. Samples were stored at 8°C until analysis.

**TABLE 2:** Optimised parameters for SFE and UHPSFC

SFx technique	UHPSFC (UPC^2^)			SFE (MV‐10)			
Injection volume	1 μL			—			
Flow rate	BSM	ISM		10 mL/min			
	1 mL/min	0.4 mL/min					
Solvent A	Carbon dioxide (CO_2_)	0.1% ammonia in methanol/water (95:5)		CO_2_			
Solvent B	Methanol	—		Methanol			
Gradient	Time (minutes)	% A	% B	Time (minutes)	% A	% B	Mode
	0	100	0	5	60	40	Dynamic
	0.2	89	11	10	60	40	Static
	2	89	11	15	60	40	Dynamic
	3	77.5	22.5	Six cycles			
	4	50	50				
	5	50	50				
	5.1	100	0				
	6	100	0				
Column	Acquity charged surface hybrid fluoro‐phenyl (3.0 mm × 100 mm, 1.7 μm)			—			
Oven temperature	40°C			85°C			
Backpressure	2100 psi			3626 psi			
PDA	220 nm			—			
Equilibration time	2 minutes			3 minutes			
QDa ESI settings							
SIR in positive mode	314.00/350.33/446.36/493.25 Da			—			
SIR in negative mode	136.99/299.00 Da			—			
Probe temperature	500°C			—			
Capillary voltage (positive and negative)	0.8 kV			—			
Cone voltage (positive and negative)	15 V			—			

SFE, supercritical fluid extraction; UHPSFC, ultra‐high‐performance supercritical fluid chromatography; SFx, supercritical fluid‐based; UPC^2^, ultra‐performance convergence chromatography; BSM, binary solvent manager; ISM, isocratic solvent manager; PDA, photodiode array; QDa, Quadrupole Dalton; ESI, electrospray ionisation; SIR, selected ion recording.

### 2.3 Analytical UHPSFC

An analytical method, the ultra‐high‐performance supercritical fluid chromatography (UHPSFC) instrument Acquity UPC^2^ (ultra‐performance convergence chromatography) comprising a sample‐, binary solvent‐, column‐, isocratic solvent‐ and convergence‐manager with a photodiode array (PDA) detector and a Quadrupole Dalton (QDa) detector was used. Nitrogen served as nebulising gas for QDa operation. The instrument was controlled via Empower 3 software. The parameters resulting in the best separation are given in Table 2. For method validation and quantitation experiments, data were collected by selected ion recording (SIR) in accordance with the specific masses of target compounds.

### 2.4 Method validation

The optimised methods for the extraction and analysis were validated in accordance to ICH (International Council for Harmonisation of Technical Requirements for Pharmaceuticals for Human Use) guidelines.^31^ Initially, a dilution series of the seven standards was prepared to obtain calibration curves and determine the linearity range. A stock solution in a concentration of 1 mg/mL (level 0) in methanol was serially diluted in a ratio of 1:3 for nine further calibration levels. Each level was injected in triplicate and the peaks were integrated using Empower 3 software. The limit of detection (LOD) and the limit of quantitation (LOQ) were determined visually by the concentration showing a signal‐to‐noise ratio of at least 3 and 10 times, respectively. Precision was determined by intraday (evaluation within 1 day) and interday (evaluation over 3 days) experiments with sample RR29. For accuracy, recovery rates were measured by spiking sample RR05 with high (125%), medium (100%) and low (75%) amounts of the respective standard compound. Spiked samples were then extracted using the MV‐10 device and analysed on the UPC^2^ as described earlier. The validation parameters are presented in Table 3.

**TABLE 3:** Results of method validation

Compounds	1	2	3	4	5	6	7
Regression equation (*y*)	(3.80 × 10^7^)*x* − 5.72 × 10^4^	(2.35 × 10^8^)*x* − 1.45 × 10^5^	(1.14 × 10^9^)*x* + 4.57 × 10^5^	(754 × 10^7^)*x* + 2.24 × 10^5^	(5.06 × 10^8^)*x* – 4.49 × 10^5^	(9.76 × 10^8^)*x* – 2.12 × 10^6^	(1.75 × 10^9^)*x* – 2.20 × 10^6^
Correlation coefficient (*R* ^2^)	0.9992	0.9991	0.9979	0.998	0.9968	0.9994	0.9911
Linearity range (μg/mL)	0.56–1,227.50	0.53–127.86	0.15–108.88	0.49–1,080.00	0.50–366.08	0.15–965.46	0.17–40.74
LOD (μg/mL)	1.12	0.33	0.08	0.25	0.25	0.15	0.047
LOQ (μg/mL)	1.9	1.19	0.23	1.48	0.76	0.67	0.17
Precision (%)							
Intraday	2.85	2.99	0.84	0.40	2.52	3.03	1.83
Interday	3.83	2.20	2.63	1.83	3.90	4.55	5.17
Accuracy (%)							
High spike	102.3	96.6	n.d.	n.d.	n.d.	n.d.	n.d.
Medium spike	99.2	102.4	n.d.	n.d.	n.d.	n.d.	n.d.
Low spike	102.4	97.7	n.d.	n.d.	n.d.	n.d.	n.d.
Combined uncertainty *U* (%)	2.55	8.10	1.52	1.50	3.09	3.18	3.87
Expanded uncertainty *U*exp (%) (*k* = 2)	5.09	16.20	3.04	2.99	6.17	6.36	7.75

LOD, limit of detection; LOQ, limit of quantitation; n.d., not determined.

In six herbal drug samples, namely RR19, RR27 and RR28–RR31, compound **3** was outside the linearity range due to its high content. Therefore, the respective samples were diluted with methanol in a ratio of 1:1^32^ in order to be inside the linear range of the validated method (Table S1).

Assessment of global uncertainty was carried out on the basis of Konieczka and Namiésnik^33^ and Ratola *et al*.^34^ Combined uncertainty (*U*) was calculated from following the expression for each compound, respectively: *U* =  √ (*U*1^2^+*U*2^2^+*U*3^2^+*U*4^2^+*U*5^2^) where *U*1 is uncertainty associated with sample preparation, *U*2 is uncertainty associated with calibration, *U*3 is uncertainty associated with precision, *U*4 is uncertainty associated with accuracy (not included for compounds **3** and **7**) and *U*5 is uncertainty associated with analyte concentration. Expanded uncertainty (*U*exp) is expressed as twice *U* (*k* = 2) (Table 3).

## 3 RESULTS AND DISCUSSION

### 3.1 Method development for extraction

With the aim to establish a SFx protocol, supercritical CO_2_ was used for both the extraction and analysis of compounds **1** to **7**. Since methanol is known as optimum solvent to extract secondary metabolites of rose root, it was used as modifier to adjust the polarity of supercritical CO_2_. Initially, a stepwise extraction using 100%, 90%, 80%, 70%, 60% and 50% CO_2_ was selected. Extraction with a mixture of 60% CO_2_ and 40% methanol as modifier revealed to be the best suitable combination to cover a broad polarity spectrum of constituents. Furthermore, parameters like temperature (60°C, 70°C, 80°C, 85°C), backpressure [active backpressure regulator (ABPR) set to 2176 psi, 2901 psi, or 3626 psi] and duration of dynamic and static mode were tested to optimise the extraction yield. One of the generated extracts (RRSFE5) of sample RR29 was then used for UHPSFC method development (parameters: 60% CO_2_, 40% methanol, flow rate 10 mL/min, oven temperature 85°C, ABPR set to 2176 psi, 5 minutes dynamic mode–5 minutes static mode–5 minutes dynamic mode, two cycles). Extraction efficiency was determined for sample RR29 with the finally optimised UHPSFC method. Since all seven compounds showed an extraction efficiency of over 95%, the final extraction method was set to 15 minutes in six cycles to ensure exhaustive extraction (Table 2).

### 3.2 UHPSFC method development

In order to separate the seven rose root constituents, method development was exemplarily carried out with the extract of sample RR29 in four steps: (i) column screening with a generic gradient followed by co‐solvent screening, (ii) optimisation of parameters like additives, back pressure, flow rate and column temperature, (iii) gradient optimisation to obtain a fast and efficient separation, and (iv) development of parameters for mass detection including selection of make‐up solvent for ionisation.

For column screening, eight different column chemistries with identical dimensions (3.0 mm × 100 mm) were tested using a generic gradient from 0 to 50% methanol within 4 minutes. The stationary phases included four of the Waters Torus series, i.e. 1‐AA (1.7 μm), DEA (1.7 μm), DIOL (1.7 μm) and 2‐PIC (1.7 μm) and four of the Waters Viridis series, i.e. BEH (1.7 μm), BEH 2‐EP (1.7 μm), CSH FP (1.7 μm) and Silica 2‐EP (5 μm). The charged surface hybrid fluoro‐phenyl (CSH FP) column resulted as the most suitable stationary phase (Supporting Information Figure S1).

The test of four different co‐solvents (methanol, ethanol, isopropyl alcohol and acetonitrile) revealed that methanol without any additives was the best choice regarding peak shape, retention time, and resolution (Figure S2). Neither the addition of acid (0.1% formic acid) nor a mixture of methanol and acetonitrile (50:50) as co‐solvent improved the result (data not shown).

A parameter unique to SFx technologies is the backpressure controlled by the ABPR. In comparison to the standard setting of 2000 psi, an improved peak shape was observed for the ABPR set to 2100 psi. A further increase of backpressure (up to 2500 psi) did not improve resolution or peak shape but only led to a retention time shift. Furthermore, the best separation was achieved with a flowrate of 1.0 mL/min and a column temperature of 40°C.

The gradient of the binary mobile phase comprising CO_2_ and methanol as co‐solvent was tested with and without isocratic intermediate steps. With the finally optimised method conditions, a separation of rose root compounds in less than 3.5 minutes was achieved (Table 2).

To ensure optimal ionisation for mass analysis using a QDa detector, a solvent composition for the isocratic solvent manager (ISM) – a post‐column solvent delivery module – was developed. Four different ISM solvents were tested: methanol/water (95:5), 0.1% formic acid in methanol/water (95:5), 0.1% ammonia in methanol/water (95:5) and 10 mM ammonium formate in methanol/water (95:5). Best results were obtained with 0.1% ammonia in methanol/water (95:5) and an ISM flow rate of 0.4 mL/min (Figure S3).

To record both the negative and the positive ionisation mode, polarity switching was employed. For each compound a distinct *m/z* value was chosen for SIR, as given in Table 2. In the positive mode, SIR was performed for [M + NH_4_]^+^ ions: 314.00 Da (**2**), 350.33 Da (**3**), 446.36 Da (**5** and **6**) and 493.25 Da (**7**). In negative mode, SIR was performed for [M − H]^−^ ions: 136.99 Da (**1**) and 299.00 Da (**4**). Both positive and negative mode showed the best ionisation results with a cone voltage of 15 V. Other parameters like capillary voltage (0.8 kV), probe temperature (500°C), sampling rate (five) and gain (three) gave the best results with default settings (Table 2).

**FIGURE 2:**
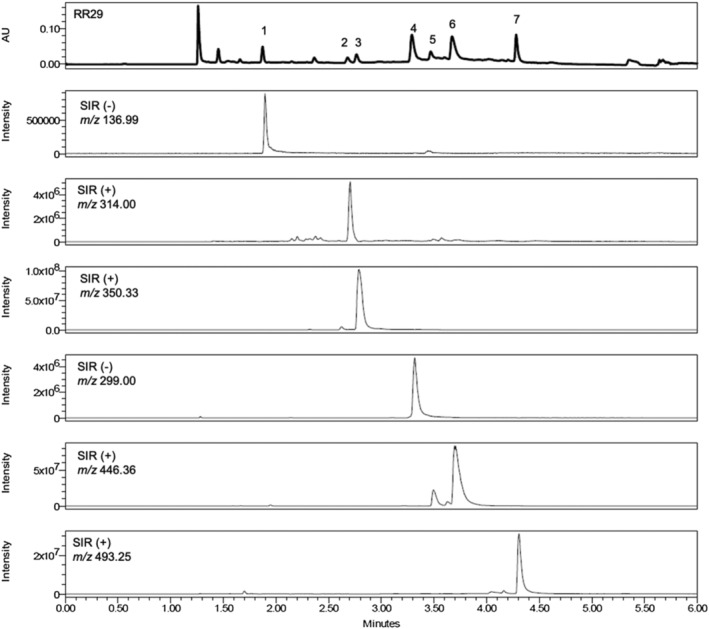
UHPSFC analysis of the herbal drug sample RR29 (PDA 220 nm) and extracted SIR chromatograms of compounds **1**–**7** with optimized parameters on a CSH FP column (1.7 μm, 100 mm × 3 mm)

The parameters for the final methods for extraction and separation are given in Table 2. The chromatogram of the extract of sample RR29 and extracted SIR channels of analytes **1**–**7** are given in Figure 2.

### 3.3 Method validation

To ensure the suitability of the generated protocols for the quantitation of compounds **1**–**7** in *R. rosea* samples using mass detection, a validation according to ICH guidelines was performed.^31^ Results shown in Table 3 including evaluation of linearity, LOD, LOQ, as well as precision and accuracy are in accordance with the ICH recommendation.

By triplicate injection of the seven analytes in serial dilutions of increasing concentrations, calibration curves were obtained with a calculated linearity ranging from 0.15 μg/mL to 1227.50 μg/mL. Linearity ranges for **1** and **4**, both compounds of the phenylethanoid‐type, cover a particularly broad concentration range of four‐orders of magnitude. This relates to the measurement of this compound class in negative mode and a stronger ionisation due to the alkaline character of the ISM solvent as compared to compounds **2**, **3**, **5**–**7** analysed in positive mode. In comparison to linearity ranges of published methods, i.e. 15.6–500.0 μg/mL,^16^ 5–700 μg/mL^19^ and 50–800 μg/mL^17^ the established SFx protocol using mass detection considerably extends the concentration range to quantify rose root secondary metabolites. This is especially valuable since plant samples often show a high variability of constituents. Linear regression analysis showed a good correlation for all standards (correlation coefficients *R* ^2^ > 0.991).

Precision was determined as standard deviation based on peak area within 1 day (intraday) and 3 days (interday) using sample RR29. Relative standard deviations (RSDs) range from 0.40% to 3.03% in intraday experiments and 1.83% to 5.17% in interday analyses. Intraday and interday variations are acceptable and typical for plant material showing some inhomogeneity, however suggesting a good precision in comparison to other published methods for phenylethanoids and phenylpropanoids.^16^, ^17^

For the determination of accuracy, sample RR05 was spiked with 125%, 100% and 75% of two representative standard compounds, namely *p*‐tyrosol (**1**) and rosin (**2**). The spiked samples were extracted and analysed as described in the established SFx protocol. Good recovery percentages ranging from 96.6% to 102.4% were found, which are in agreement with published chromatographic methods.^18^

To ensure measurement reliability, combined and expanded uncertainties (*U* and *U*exp) were calculated. In fact, *U* resulted in a value below 5% for compounds **1** and **3**–**7**. The highest *U* value was calculated for compound **2** (*U* = 8.10%). This can be deduced from a high standard deviation, a relatively narrow linearity range compared to the other compounds and a broader dispersion of recovery rates compared to phenylethanoids (**1**). Hence, compound **2** results in a higher uncertainty value. Overall *U* and *U*exp are acceptable and in accordance with published literature.^33^

### 3.4 Analysis of samples

All seven analytes were quantitated in a total of 31 samples (RR01–RR31): Seven commercial samples (RR01–RR07), including two herbal medicinal products (RR01 and RR02) registered in Austria, and 24 herbal drugs (RR08–RR31) were analysed (Table S1).

**FIGURE 3:**
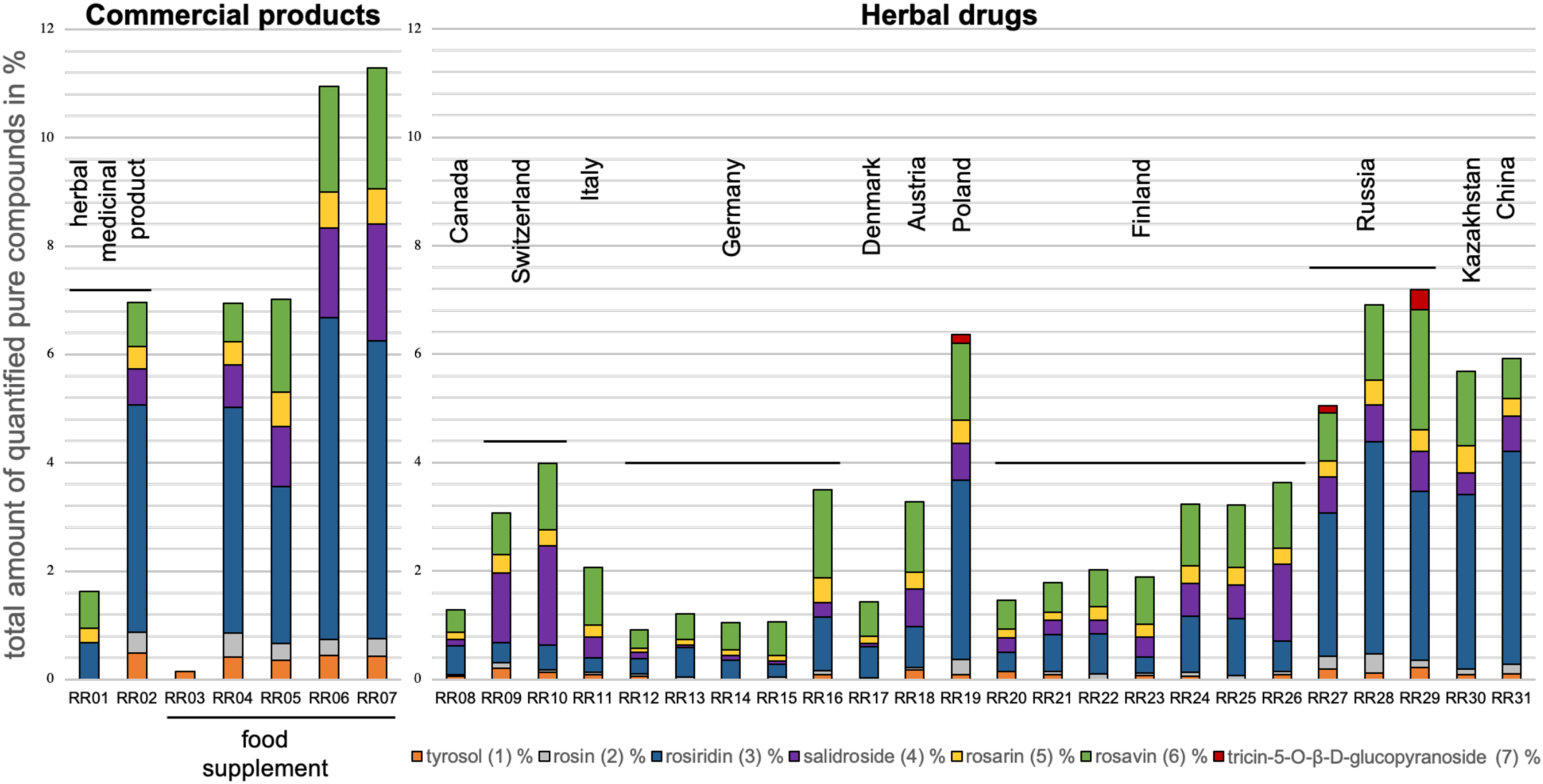
Total amount (%) of all seven analytes (**1**–**7**) in seven commercial products and 24 herbal drugs

Extracts of *R. rosea* roots and rhizomes are mostly sold in the form of tablets or capsules for oral administration. To date in Europe, only the Herbal Medicinal Products Committee (HMPC) provides a monograph for *R. rosea* that requires a drug‐to‐extract ratio 1.5–5:1 using 67–70% ethanol as extraction solvent (EMA/HMPC/232091/2011). However, these directions give room for a broad range of metabolites present in the extract, as can be seen from our quantitative analysis of sample RR01 and RR02 (Figure 3). Although both products fulfil the HMPC requirement, *p*‐tyrosol (**1**), rosin (**2**) and salidroside (**4**) could not be detected in RR01, whereas RR02 contains 6.95% of the quantified compounds in total. RR03 only contains 0.15% *p*‐tyrosol (**1**) and none of the other six standard compounds was detected, while the other four samples (RR04–RR07) are similar to the herbal medicinal product RR02 or even higher in total secondary metabolite content. Some manufacturers declare a minimum content of salidroside (**4**) and/or total rosavins (the sum of **2**, **5** and **6**): RR04 claims to contain 5.27% rosavins and 2.36% salidroside; RR05 should contain 3% rosavins and RR06 declares 3% rosavins and 1% salidroside. The USP monograph requires a range between 90% and 110% of the declared content of rosavins and salidroside.^35^ Only one sample – RR06 – with a quantified amount of 2.91% of rosavins is in accordance with these USP requirements. However, the salidroside content in RR06 exceeds its declaration of 1% salidroside (1.66% quantified) and is therefore outside the limit given by the USP monograph. In conclusion, none of the analysed commercial products complies with its declared content of constituents.

The results of the quantitation of the investigated herbal drugs are given in Figure 3, ordered by origin from West to East. In general, there is a high variability of the quantified amount of the individual compounds **1**–**7** as well as in their total content. The high total metabolite content of Asian samples is immediately apparent, i.e. samples from Russia (RR27–RR29), Kazakhstan (RR30) and China (RR31). Since additional information (Table S1) concerning the organ of the cultivated samples was available for the samples from Finland (RR20–RR26), roots and rhizomes could be compared: samples containing exclusively roots (RR22 and RR23) showed a significantly lower content of quantified compounds than rhizome samples (RR24–RR26) which is in accordance to previous studies.^14^, ^15^

Compounds **3**, **4** and **6** are the most abundant constituents in herbal drug samples: rosiridin (**3)** accounts as the major compound in samples RR19, RR27 and RR29–RR31; salidroside (**4**) was detected with the highest content in Swiss samples RR09 and RR10 and rosavin (**6**) has the highest amount in samples RR11, RR15–RR18 and RR23–RR25.

Tricin‐5‐*O*‐β‐d‐glucopyranoside (**7**) was detected only in three samples derived from Poland (RR19) and Russia (RR27 and RR29).

Although the first applications of SFx technologies were already reported more than 50 years ago, this is the first time, an SFx protocol was established for rose root constituents. A reproducible, precise, and accurate protocol for the fast extraction and quantitation of seven secondary metabolites was generated. This proves once more the high potential and applicability of environmentally friendly and robust CO_2_ based instruments not only for non‐polar but also for polar constituents. Using supercritical fluids for both, chromatography and targeted extraction (SFE), allows for a very focused and straightforward procedure without the need for any specific sample clean‐up prior to analysis. In addition, the selectivity, reliability, and separation speed of the UHPSFC technique even exceeds classical methods like HPLC, UPLC, and gas chromatography (GC).^16^, ^18^, ^19^, ^21^ As presented here, the hyphenation of a UHPSFC device to a mass detector (QDa) provides additional benefits such lower detection limits than conventional set‐ups.

Considering the rising popularity of *Rhodiola* commodities, fast and reliable methods to analyse the content of key constituents are of great importance to guarantee the supply of high‐quality products. Therefore, and to test the versatility of SFx techniques, we aimed not only to provide a workflow for the extraction and quantitation of well‐established marker compounds (e.g. rosavins) but also of minor polar constituents (e.g. tricin‐5‐*O*‐β‐d‐glucopyranoside). The presented quantitative analyses of 24 drug substances and seven commercial products of *R. rosea* revealed substantial variabilities of their metabolite profile. Most astonishingly, none of the analysed commercial samples met the content of salidroside and/or rosavins declared on the package.

Without having properly authenticated materials, it can only be observed that samples cultivated in Asia (China, Kazakhstan and Russia) resulted in a high overall content of quantified metabolites which may be the result of harsh environmental conditions stimulating the plants' chemical defence machinery.^1^, ^13^, ^36^ The sample from Poland (RR19) which additionally contains tricin‐5‐*O*‐β‐d‐glucopyranoside (**7**) is comparable to the Asian samples.

Compared to the USP, the Russian Pharmacopoeia requires higher minimum contents for roots and rhizomes of *R. rosea*, i.e. 1.0% rosavins and 0.8% salidroside (Table 1). Taking this into account when comparing the herbal drug samples RR08–RR31, only RR09 and RR10 originating from Switzerland as well as RR26 from Finland meet these requirements. Surprisingly, three out of the five food supplement samples RR03–RR07, i.e. RR05, RR06, and RR07, also meet these comparably high contents required by the Russian Pharmacopoeia for rosavins and salidroside.^12^ When considering the USP monograph for *R. rosea*, all herbal samples except for RR15 and RR17 fulfil the requirements (> 0.3% rosavins and > 0.08% salidroside).^9^ The same accounts for commercial products except for the herbal medicinal product RR01, where no salidroside was detected and the food supplement RR03, where neither salidroside nor rosavins were found.

Comparing the quantities of *R. rosea* constituents determined by the SFx workflow established in the present study with results from classic quantitation methods reported in the scientific literature, similar concentration ranges were found (Table S1). The high variations of constituent concentrations in samples from different origins have also been observed in previous quantitative analyses.^16^, ^18^, ^19^

In sum, we have demonstrated that SFx technologies are suitable for the extraction and quantitation of *Rhodiola* constituents in various samples. Whether the here developed SFx protocol is also suitable for the authentication of *Rhodiola* raw material and if it can be applied to distinguish between different *Rhodiola* species and potential adulterations needs to be investigated in future studies.

## CONFLICT OF INTEREST

The authors declare no conflict of interest.

## Supporting information


**Figure S1.** Column screening using the SFE extract of sample RR29 with methanol as co‐solvent (PDA at 220 nm): BEH (1.7 μm), BEH 2‐EP (1.7 μm), CSH FP (1.7 μm), Silica 2‐EP (5 μm), 1‐AA (1.7 μm), 2‐PIC (1.7 μm), DEA (1.7 μm), and DIOL (1.7 μm). Column dimensions: 100 mm × 3 mm
**Figure S2.** Co‐solvent screening using the SFE extract of sample RR29 with the CSH FP (1.7 μm) column. Column dimensions: 100 mm × 3 mm, PDA at 220 nm
**Figure S3.** Influence of different make‐up solvents on the ionisation in negative and positive mode (TIC): (i) mixture of 95% methanol and 5% water, (ii) mixture of 95% methanol and 5% water with 0.1% formic acid, (iii) mixture of 95% methanol and 5% water with 0.1% ammonia and (iv) mixture of 95% methanol and 5% water with 10 mM ammonium formate
**Table S1.** Percentage of rose root secondary metabolites (**1**–**7**) in investigated samples (RR01–RR31) (*n* = 3) determined using the SIR signals at the respective *m/z* value for quantitation
**Table S2.** Detail information of investigated samples RR01–RR31 including sample type, origin, declared content, organ and batch number
