# Supplementary material for: A supercritical fluid workflow for the quality assessment of herbal drugs and commercial preparations from Rhodiola rosea
Source: Phytochem Anal. 2021 Feb 26;32(6):982–91. doi: 10.1002/pca.3040 (PMC8596633; doi:10.1002/pca.3040)
Supplement: Supplementary file 1 — Figure S1. Column screening using the SFE extract of sample RR29 with methanol as co‐solvent (PDA at 220 nm): BEH (1.7 μm), BEH 2‐EP (1.7 μm), CSH FP (1.7 μm), Silica 2‐EP (5 μm), 1‐AA (1.7 μm), 2‐PIC (1.7 μm), DEA (1.7 μm), and DIOL (1.7 μm). Column dimensions: 100 mm × 3 mm Figure S2. Co‐solvent screening using the SFE extract of sample RR29 with the CSH FP (1.7 μm) column. Column dimensions: 100 mm × 3 mm, PDA at 220 nm Figure S3. Influence of different make‐up solvents on the ionisation in negative and positive mode (TIC): (i) mixture of 95% methanol and 5% water, (ii) mixture of 95% methanol and 5% water with 0.1% formic acid, (iii) mixture of 95% methanol and 5% water with 0.1% ammonia and (iv) mixture of 95% methanol and 5% water with 10 mM ammonium formate Table S1. Percentage of rose root secondary metabolites (1–7) in investigated samples (RR01–RR31) (n = 3) determined using the SIR signals at the respective m/z value for quantitation Table S2. Detail information of investigated samples RR01–RR31 including sample type, origin, declared content, organ and batch number [file PCA-32-982-s001.docx]

**Supporting Information**

**A supercritical fluid workflow for the quality assessment of herbal drugs and commercial preparations from *Rhodiola rosea***

Julia Langeder^a^ (ORCID 0000-0002-1991-9274) and Ulrike Grienke^a^ (ORCID 0000-0003-0305-9270)

**Affiliation**

^a^ Department of Pharmacognosy, University of Vienna, Vienna, Austria

**Correspondence**

Univ.-Ass. Mag. pharm. Dr. Ulrike Grienke, Department of Pharmacognosy, Faculty of Life Sciences, University of Vienna, Althanstraße 14, 1090 Vienna, Austria.

E-mail: [Ulrike.Grienke@univie.ac.at](mailto:Ulrike.Grienke@univie.ac.at) Phone: +43 1 4277 55262 FAX: +43 1 4277 855262

**Figure S1** Column screening using the SFE extract of sample RR29 with MeOH as co-solvent (PDA at 220 nm): BEH (1.7 µm), BEH 2-EP (1.7 µm), CSH FP (1.7 µm), Silica 2-EP (5 µm), 1-AA (1.7 µm), 2-PIC (1.7 µm), DEA (1.7 µm), and DIOL (1.7 µm). Column dimensions: 100 mm x 3 mm


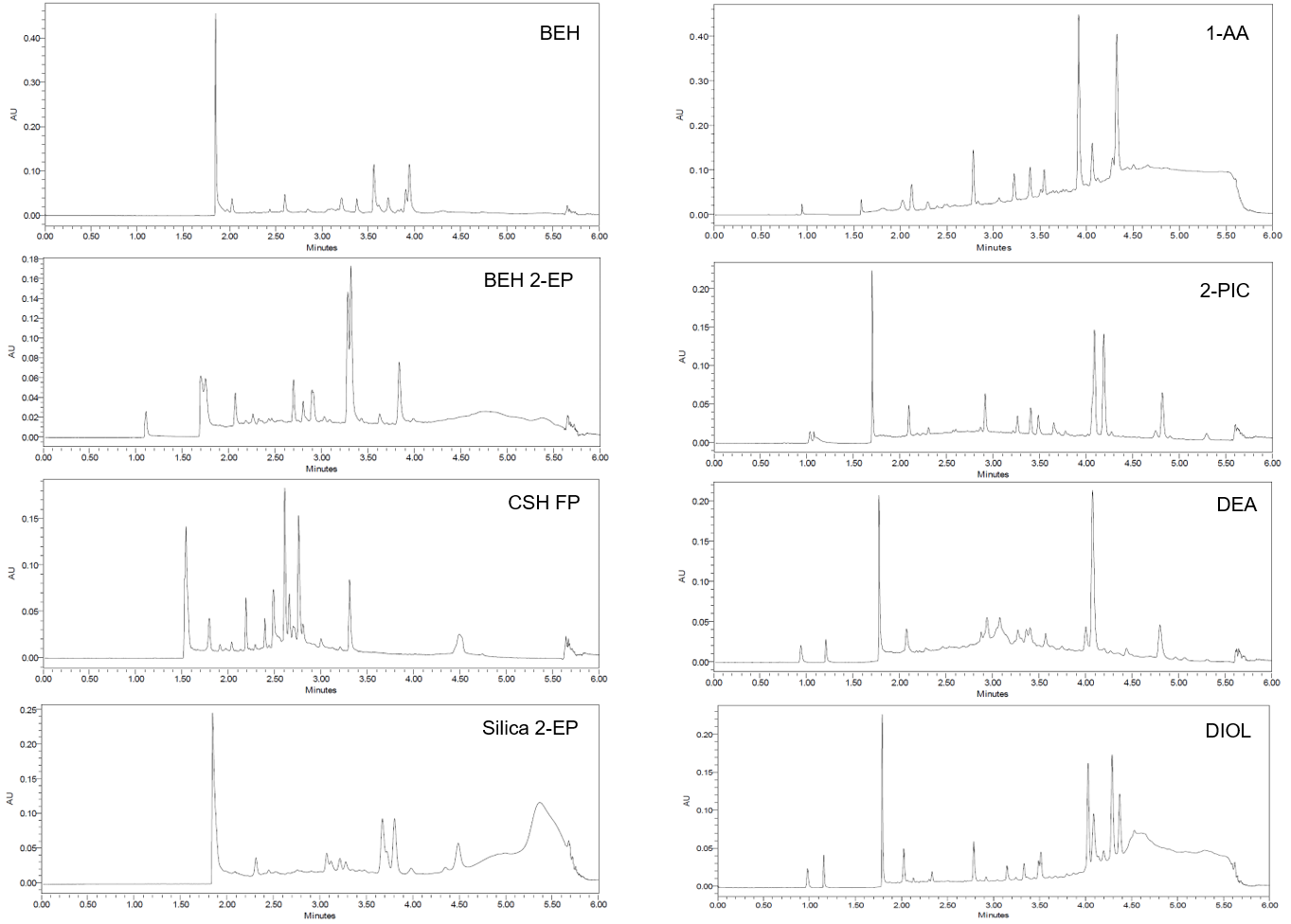


**Figure S2** Co-solvent screening using the SFE extract of sample RR29 with the CSH FP (1.7 µm) column. Column dimensions: 100 mm x 3 mm, PDA at 220 nm


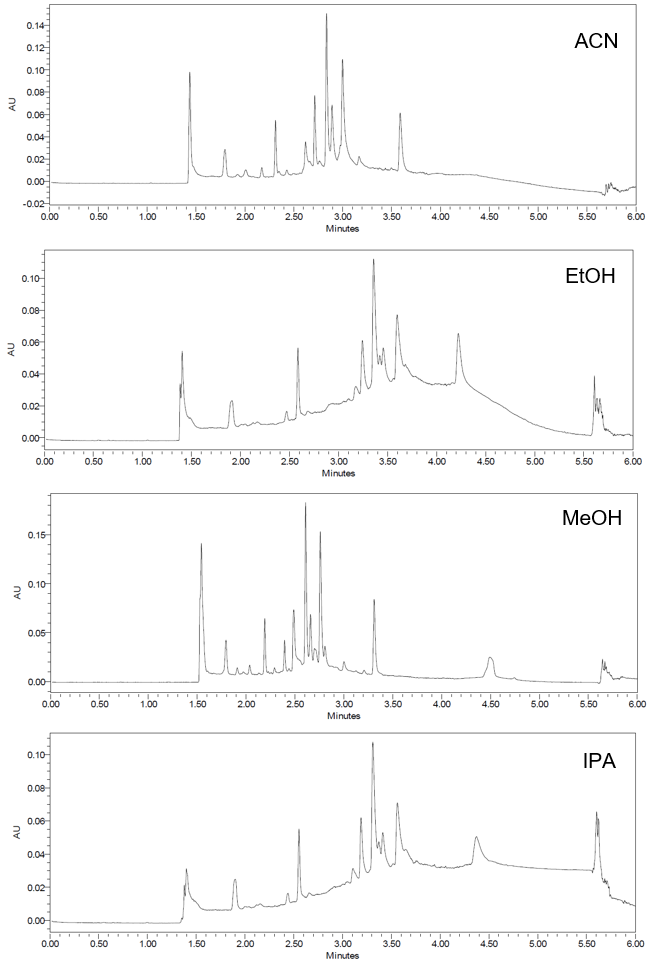


**Figure S3** Influence of different make-up solvents on the ionization in negative and positive mode (TIC): (i) mixture of 95% MeOH and 5% H_2_O, (ii) mixture of 95% MeOH and 5% H_2_O with 0.1% formic acid, (iii) mixture of 95% MeOH and 5% H_2_O with 0.1% NH_3_ and (iv) mixture of 95% MeOH and 5% H_2_O with 10 mM ammonium formate


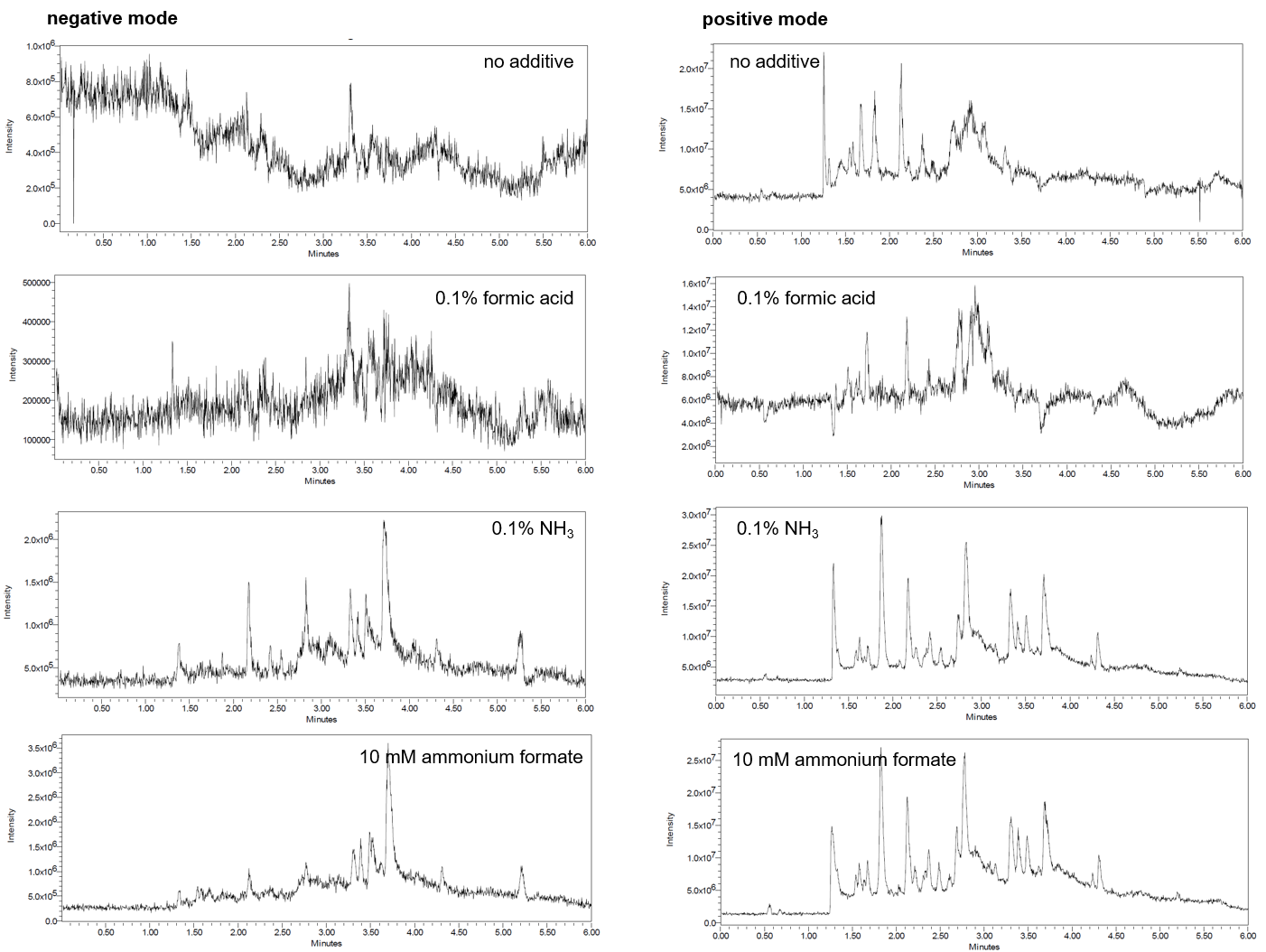


**Table S1** Percentage of rose root secondary metabolites (**1**-**7**) in investigated samples (RR01-31) (n = 3) determined using the SIR signals at the respective *m/z* value for quantitation

| **Sample name** | **Tyrosol**  **(1)** | | **Rosin**  **(2)** | | **Rosiridin**  **(3)** | | **Salidroside**  **(4)** | | **Rosarin**  **(5)** | | **Rosavin**  **(6)** | | **Tricin-5-*O*-β-D-**  **glucopyranoside**  **(7)** | |
| --- | --- | --- | --- | --- | --- | --- | --- | --- | --- | --- | --- | --- | --- | --- |
|  | % | RSD % | % | RSD % | % | RSD  % | % | RSD  % | % | RSD % | % | RSD % | % | RSD  % |
| Liter-ature data | 0.05-0.93^a^ |  | 0.01-0.66^a^ |  | - |  | 1.01-13.2^a^ |  | 0.13-1.09^a^ |  | 0.03-2.71^a^ |  | - |  |
|  | - |  | 0.02-0.08^b^ |  | 0.07-0.38^b^ |  | 0.04-0.27^b^ |  | 0.02-0.11^b^ |  | 0.06-0.35^b^ |  | - |  |
|  | - |  | 0.01-0.42^c^ |  | - |  | 0.08-0.40^c^ |  | 0.04-0.47^c^ |  | 0.01-1.58^c^ |  | - |  |
| RR01 | **n.d.**^d^ | n.d. | **n.d.** | n.d. | **0.67** | 0.70 | **n.d.** | n.d. | **0.28** | 3.43 | **0.67** | 0.47 | **n.d.** | n.d. |
| RR02 | **0.48** | 2.60 | **0.38** | 2.11 | **4.21** | 0.54 | **0.66** | 4.47 | **0.41** | 4.10 | **0.81** | 3.63 | **n.d.** | n.d. |
| RR03 | **0.15** | 2.55 | **n.d.** | n.d. | **n.d.** | n.d. | **n.d.** | n.d. | **n.d.** | n.d. | **n.d.** | n.d. | **n.d.** | n.d. |
| RR04 | **0.41** | 3.27 | **0.44** | 3.50 | **4.17** | 0.44 | **0.78** | 3.78 | **0.43** | 3.96 | **0.71** | 4.71 | **n.d.** | n.d. |
| RR05 | **0.35** | 3.20 | **0.31** | 1.41 | **2.89** | 0.81 | **1.11** | 2.26 | **0.65** | 2.91 | **1.70** | 1.24 | **n.d.** | n.d. |
| RR06 | **0.43** | 4.65 | **0.30** | 1.34 | **5.94** | 2.16 | **1.66** | 3.58 | **0.67** | 2.93 | **1.94** | 3.64 | **n.d.** | n.d. |
| RR07 | **0.43** | 2.18 | **0.32** | 3.36 | **5.50** | 1.28 | **2.17** | 3.99 | **0.64** | 0.49 | **2.23** | 2.82 | **n.d.** | n.d. |
| RR08 | **0.05** | 4.03 | **0.04** | 1.53 | **0.53** | 0.57 | **0.12** | 0.95 | **0.13** | 2.92 | **0.42** | 2.29 | **n.d.** | n.d. |
| RR09 | **0.21** | 0.37 | **0.10** | 1.91 | **0.37** | 0.19 | **1.28** | 0.42 | **0.33** | 1.17 | **0.76** | 0.96 | **n.d.** | n.d. |
| RR10 | **0.14** | 3.82 | **0.04** | 3.29 | **0.46** | 0.43 | **1.82** | 0.41 | **0.30** | 3.17 | **1.23** | 2.42 | **n.d.** | n.d. |
| RR11 | **0.09** | 2.13 | **0.04** | 2.08 | **0.26** | 1.05 | **0.38** | 2.56 | **0.23** | 0.29 | **1.05** | 0.88 | **n.d.** | n.d. |
| RR12 | **0.06** | 4.55 | **0.05** | 2.70 | **0.28** | 0.18 | **0.11** | 1.20 | **0.08** | 3.02 | **0.35** | 2.98 | **n.d.** | n.d. |
| RR13 | **n.d.** | n.d. | **0.04** | 2.81 | **0.56** | 0.21 | **0.04** | 3.89 | **0.10** | 3.02 | **0.48** | 2.79 | **n.d.** | n.d. |
| RR14 | **n.d.** | n.d. | **n.d.** | n.d. | **0.36** | 0.35 | **0.09** | 1.26 | **0.11** | 3.22 | **0.50** | 1.71 | **n.d.** | n.d. |
| RR15 | **n.d.** | n.d. | **0.04** | 2.55 | **0.24** | 0.24 | **0.07** | 1.02 | **0.10** | 1.44 | **0.63** | 1.17 | **n.d.** | n.d. |
| RR16 | **0.09** | 3.10 | **0.07** | 2.35 | **0.99** | 0.34 | **0.26** | 3.84 | **0.46** | 1.51 | **1.62** | 1.81 | **n.d.** | n.d. |
| RR17 | **n.d.** | n.d. | **0.03** | 3.08 | **0.56** | 0.99 | **0.07** | 2.80 | **0.14** | 3.47 | **0.62** | 0.78 | **n.d.** | n.d. |
| RR18 | **0.18** | 4.06 | **0.05** | 1.37 | **0.75** | 0.47 | **0.69** | 1.17 | **0.31** | 2.24 | **1.31** | 0.41 | **n.d.** | n.d. |
| RR19 | **0.09** | 3.83 | **0.27** | 3.55 | **3.31**^e^ | 1.90 | **0.68** | 0.79 | **0.43** | 0.61 | **1.42** | 1.95 | **0.16** | 0.40 |
| RR20 | **0.15** | 2.27 | **n.d.** | n.d. | **0.35** | 1.42 | **0.26** | 1.33 | **0.17** | 0.81 | **0.53** | 1.72 | **n.d.** | n.d. |
| RR21 | **0.09** | 4.51 | **0.06** | 4.66 | **0.69** | 2.17 | **0.26** | 2.41 | **0.15** | 1.46 | **0.54** | 1.87 | **n.d.** | n.d. |
| RR22 | **n.d.** | n.d. | **0.10** | 3.96 | **0.75** | 2.41 | **0.25** | 2.05 | **0.24** | 1.03 | **0.69** | 3.99 | **n.d.** | n.d. |
| RR23 | **0.07** | 3.34 | **0.04** | 2.57 | **0.30** | 0.68 | **0.36** | 2.62 | **0.24** | 0.77 | **0.88** | 2.77 | **n.d.** | n.d. |
| RR24 | **0.05** | 3.73 | **0.08** | 2.18 | **1.03** | 1.33 | **0.61** | 1.50 | **0.33** | 1.08 | **1.14** | 2.66 | **n.d.** | n.d. |
| RR25 | **n.d.** | n.d. | **0.07** | 2.25 | **1.04** | 0.40 | **0.63** | 2.18 | **0.32** | 0.73 | **1.15** | 2.84 | **n.d.** | n.d. |
| RR26 | **0.08** | 2.14 | **0.07** | 3.96 | **0.56** | 1.23 | **1.41** | 2.61 | **0.29** | 0.88 | **1.21** | 2.02 | **n.d.** | n.d. |
| RR27 | **0.19** | 2.02 | **0.23** | 3.34 | **2.65**^e^ | 2.65 | **0.66** | 2.27 | **0.30** | 2.48 | **0.88** | 2.54 | **0.13** | 3.26 |
| RR28 | **0.12** | 0.36 | **0.35** | 0.56 | **3.92**^e^ | 0.58 | **0.68** | 1.65 | **0.45** | 0.22 | **1.39** | 2.72 | **n.d.** | n.d. |
| RR29 | **0.21** | 0.60 | **0.14** | 4.44 | **3.11**^e^ | 0.32 | **0.74** | 0.55 | **0.40** | 1.81 | **2.21** | 0.73 | **0.36** | 3.56 |
| RR30 | **0.09** | 4.01 | **0.10** | 2.33 | **3.22**^e^ | 0.83 | **0.40** | 2.44 | **0.50** | 2.63 | **1.37** | 1.93 | **n.d.** | n.d. |
| RR31 | **0.11** | 2.81 | **0.17** | 0.54 | **3.92**^e^ | 0.35 | **0.66** | 1.46 | **0.32** | 1.20 | **0.74** | 2.64 | **n.d.** | n.d. |

^a^ Ma et al, 2011 ^b^ Ganzera et al, 2001 ^c^ Alperth et al, 2019  ^d^ not determined (below LOQ)

^e^ 1:1 dilution of sample was used to measure **3** within the linearity range

**Table S2** Detail information of investigated samples RR01-31 including sample type, origin, declared content, organ and batch number

| **Sample name** | **Sample type** | **Origin** | **Declared content** | **Organ** | **Batch/Voucher number** |
| --- | --- | --- | --- | --- | --- |
| RR01 | Herbal medicinal product | n.g.^a^ | DER^b^ 1.5-5:1. 70% ethanol | Root & rhizome | 1901667 |
| RR02 | Herbal medicinal product | n.g. | DER 1.5-5:1. 60% ethanol | Root & rhizome | 0841217 |
| RR03 | Food supplement | n.g. | - | Root & rhizome | L28261219 |
| RR04 | Food supplement | n.g. | 5.27% rosavins, 2.36% salidroside | Root & rhizome | 19AB350B |
| RR05 | Food supplement | n.g. | 3% rosavins | Root & rhizome | 429584 |
| RR06 | Food supplement | n.g. | 3% rosavins, 1% salidroside | Root & rhizome | 50169178 |
| RR07 | Food supplement | n.g. | - | Root & rhizome | L9158 |
| RR08 | Herbal drug | Canada | - | Root & rhizome | JR-20200924-D1 |
| RR09 | Herbal drug | Switzerland | - | Root & rhizome | JR-20200924-A1 |
| RR10 | Herbal drug | Switzerland | - | Root & rhizome | JR-20200924-A2 |
| RR11 | Herbal drug | Italy | - | Root & rhizome | JR-20200924-D2 |
| RR12 | Herbal drug | Germany | - | Root & rhizome | JR-20200924-E1 |
| RR13 | Herbal drug | Germany | - | Root & rhizome | JR-20200924-E2 |
| RR14 | Herbal drug | Germany | - | Root & rhizome | JR-20200924-E3 |
| RR15 | Herbal drug | Germany | - | Root & rhizome | JR-20200924-D3 |
| RR16 | Herbal drug | Germany | - | Root & rhizome | JR-20200924-D4 |
| RR17 | Herbal drug | Denmark | - | Root & rhizome | JR-20200924-E4 |
| RR18 | Herbal drug | Austria | - | Root & rhizome | JR-20200924-D5 |
| RR19 | Herbal drug | Poland | - | Root & rhizome | JR-20200924-D6 |
| RR20 | Herbal drug | Finland | - | Root & rhizome | JR-20200924-D7 |
| RR21 | Herbal drug | Finland | - | Root & rhizome | JR-20200924-B1 |
| RR22 | Herbal drug | Finland | - | Root | JR-20200924-B2 |
| RR23 | Herbal drug | Finland | - | Root | JR-20200924-C2 |
| RR24 | Herbal drug | Finland | - | Rhizome | JR-20200924-B3 |
| RR25 | Herbal drug | Finland | - | Rhizome | JR-20200924-B4 |
| RR26 | Herbal drug | Finland | - | Rhizome | JR-20200924-C1 |
| RR27 | Herbal drug | Russia | - | Root & rhizome | JR-20200924-E5 |
| RR28 | Herbal drug | Russia | - | Root & rhizome | JR-20200924-D8 |
| RR29 | Herbal drug | Russia | - | Root & rhizome | JR-20200924-F1 |
| RR30 | Herbal drug | Kazakhstan | - | Root & rhizome | JR-20200924-D9 |
| RR31 | Herbal drug | China | - | Root & rhizome | JR-20200924-D10 |

^a^ not given ^b^ drug-extraction ratio
